# Effects of Deep Tillage on Rhizosphere Soil and Microorganisms During Wheat Cultivation

**DOI:** 10.3390/microorganisms12112339

**Published:** 2024-11-16

**Authors:** Junkang Sui, Chenyu Wang, Feifan Hou, Xueting Shang, Qiqi Zhao, Yuxuan Zhang, Yongqiang Hou, Xuewen Hua, Pengfei Chu

**Affiliations:** College of Agriculture and Biology, Liaocheng University, Liaocheng 252000, China; suijunkang@lcu.edu.cn (J.S.); wcy20031214@163.com (C.W.); hff050129@163.com (F.H.); sxt031216@163.com (X.S.); zyx004724@163.com (Y.Z.); hyq0486@163.com (Y.H.); huaxuewen@lcu.edu.cn (X.H.)

**Keywords:** rhizosphere, deep tillage, microbial community, microbial function, soil fertility

## Abstract

The production of wheat is fundamentally interconnected with worldwide food security. The practice of deep tillage (DT) cultivation has shown advantages in terms of soil enhancement and the mitigation of diseases and weed abundance. Nevertheless, the specific mechanisms behind these advantages are unclear. Accordingly, we aimed to clarify the influence of DT on rhizosphere soil (RS) microbial communities and its possible contribution to the improvement of soil quality. Soil fertility was evaluated by analyzing several soil characteristics. High-throughput sequencing techniques were utilized to explore the structure and function of rhizosphere microbial communities. Despite lowered fertility levels in the 0–20 cm DT soil layer, significant variations were noted in the microbial composition of the DT wheat rhizosphere, with Acidobacteria and Proteobacteria being the most prominent. Furthermore, the abundance of Bradyrhizobacteria, a nitrogen-fixing bacteria within the Proteobacteria phylum, was significantly increased. A significant increase in glycoside hydrolases within the DT group was observed, in addition to higher abundances of amino acid and carbohydrate metabolism genes in the COG and KEGG databases. Moreover, DT can enhance soil quality and boost crop productivity by modulating soil microorganisms’ carbon and nitrogen fixation capacities.

## 1. Introduction

Wheat (*Triticum aestivum* L.) is cultured extensively worldwide, with a yearly output of over 800 million tons, of which China accounts for over 137 million tons (http://www.fao.org/faostat/ accessed on 25 July 2024). About 20% of global dietary caloric and protein intake is attributed to wheat [1]. In certain regions, the rate of fertilizer application in wheat cultivation exceeds the necessary amount [2]. Although increasing the rate of nitrogen application can enhance the protein content and compositional quality of wheat, it may also lead to its inefficient utilization and elevate the risk of environmental pollution. Boosting the nitrogen implementation rate can improve the protein and constituent compositions of wheat, thereby reducing effective utilization and heightening environmental pollution risks [3]. The prolonged application of inorganic fertilizers, which are used to improve soil fertility for agricultural production, results in soil acidification, decreased soil organic matter, and physical soil deterioration [4]. Fertility is retained in the soil above the plow bottom for extended periods, necessitating the use of novel tillage techniques and conventional planting methods to improve soil quality and maximize wheat production due to the progressive accumulation of excessive fertility. The modification of tillage techniques can optimize the interactions between soil water, gas, fertilizer, and heat; address crop–soil disputes; reduce soil nutrient and water losses; and promote regular crop growth and development [5]. Deep tillage (DT) is recognized as an effective method for improving soil characteristics and maximizing nutrient recovery [6,7].

Conventional continuous rotary tillage may develop a shallow plow layer, harden the plow bottom, accumulate soil nutrients in the surface layer, and reduce the ability to supply fertilizers to crops in the later growth stages [8]. Conversely, DT can disturb solidified plow bottoms, improve soil water permeability, and enable the comprehensive blending of nutrients across the entire plow layer. This can alleviate the limitations of nutrient accessibility that are attributed to soil compaction [9]. Moreover, DT offers advantages in terms of soil turning, loosening, mixing, and breaking, alongside the ability to enhance crop yields by using acceptable deep plowing [10]. Typically, DT is performed once to enhance the loosening of the subsurface, the infiltration of water, and the penetration of roots [11]. Although this facilitates improved nutrient uptake by crop roots, it may result in excessive nitrogen depletion [12]. By applying DT to a depth of 30 cm, the overall nitrogen concentration in the soil layer between 0 and 40 cm was increased. This, in turn, promoted better crop root dispersion, increased water efficiency, and eventually increased grain yields. A rotational tillage strategy comprising DT in the first year and no tillage in the following two years has been shown to enhance winter wheat production stability and sustainability [13].

The rhizosphere is a crucial microhabitat for plant microbiota that is situated at the interface between roots and soil [14]. Microbiological communities that exist independently within the rhizosphere participate in mutualistic interactions with plants [15], whereas soil microbial communities swiftly respond to stress, causing changes in the populations of dominant microorganisms [16]. Cultivation methods play an important role in shaping microbial community structure and influencing other environmental, ecological, and agricultural soil parameters [17]. Rotary tilling adds complexity to bacterial networks, but deeper tilling simplifies fungal networks [18]. Soil deep tillage may encourage root penetration, enhance root development, increase nutrient accumulation, and change soil microbial community structure [19].

DT procedures exert a substantial influence on soil microorganisms [20], therefore influencing the structures of microbial networks in diverse manners. Different tillage methods result in different soil microbial diversity. First, soil bacteria distribution is uneven across soil depths due to microenvironmental influences. Treatments such as DT can modify this vertical distribution [21]. Second, changes in soil fertility are another important factor, and pH is considered the main driving factor for soil bacterial communities [22]. Previous studies have confirmed that no tillage typically lowers soil pH due to the accumulation of organic matter in the surface soil [23,24]. At the same time, tillage practices may affect the distribution of aerobic, anaerobic, and facultative anaerobic bacteria [25].

Previous studies have shown that DT cultivation changes the soil layer, destroys fungal hyphae, decreases fungal richness [24,26,27], and increases the recruitment and assembly of bacteria by plant roots, contributing to a higher diversity of bacterial communities [28]. However, since most soil fungi are aerobic [29], improving aeration conditions in deeply cultivated soil will be beneficial for fungal growth. The impact of cultivation methods on soil fungal communities is mediated by soil nutrient status [30]. Soil AP and AK are considered important driving factors for fungal communities [21].

Accordingly, we explored the influence of DT on the composition and functionality of the microbial community in the wheat rhizosphere. Additionally, we aimed to clarify the fundamental mechanisms by which DT impacts wheat planting.

## 2. Methods

### 2.1. Plant Material and Treatment

This study was performed in an experimental field located in the contracted land of Liaocheng Chuangju Fengwanjiang Agricultural Technology Development Co., Ltd. (115.77° E, 36.53° N) in Dongchangfu District, Liaocheng, China. Liaocheng is characterized by a semi-dry continental climate within a warm temperate monsoon climate zone. This region has an appropriate climate: ample sunlight, with 2463.0–2741.8 annual sunshine hours; an average temperature of 12.8–13.4 °C; annual precipitation levels of 567.7–637.3 mL; an average relative humidity of 56–68%; and a frost-free period of nearly 200 days, defined by dominant southerly and slightly southerly winds. The soil in the experimental plot is sandy loam soil which was provided by Data Center for Resources and Environmental Sciences, Chinese Academy of Sciences (RESDC) (http://www.resdc.cn accessed on 12 October 2024). The soil was subjected to DT with a depth of up to 30 cm before wheat was sown in October 2022, while for the control group, it was at a depth of 15 cm, which are common depths for no-deep tillage. The application of efficient water and fertilizer management techniques was imperative throughout the wheat-growing season. Timely irrigation and application of corresponding fertilizers were carried out during the sowing period, tillering period, jointing period, and filling period. Apart from the variations in tillage depths, the DT and control groups maintained uniformity in planting methods, water and fertilizer management, as well as pest and disease control measures.

### 2.2. Sampling and Soil Fertility Measurement

The root samples were collected on 27 May 2023, at a depth of 15–20 cm. The bulk oils (non-rhizosphere soil) of the root system were shaken off in the clean bench, and the soil attached to the roots with a thickness of about 1 mm was defined as the rhizosphere soil [31]. The root samples were transferred to a sterile 50 mL centrifuge tube containing 20 mL sterile 10 mM PBS solution, placed on a full temperature shaker at 120 rpm/min, and shaken at room temperature for 20 min [32,33]. The roots were picked out of the 50 mL centrifuge tube using sterile forceps, and the remaining suspension was centrifuged at high speed (6000× *g*, 4 °C) for 20 min to collect the rhizosphere soil [34]. Three representative replicates for each group were obtained using simple random sampling. For the subsequent high-throughput sequencing and soil fertility analysis, rhizosphere soil samples were taken from DT and non-DT wheat (CK). We stored the samples at −80 °C after quick freezing in liquid nitrogen.

Soil total nitrogen (TN), phosphorus (TP), and potassium (TK), as well as available phosphorus (AP) and potassium (AK), were analyzed using sulfuric acid digestion and Kjeldahl nitrogen determination [35]; NaOH alkali melting molybdenum–antimony spectrophotometry; NaOH alkali fusion flame photometry; sodium bicarbonate/sodium fluoride hydrochloric acid extraction; the molybdenum–antimony colorimetric method [36,37]; and ammonium acetate extraction flame photometry, respectively [38]. In addition, we quantified soil nitrate nitrogen (NN) content via potassium chloride solution extraction and dual-wavelength colorimetry, and ammonium nitrogen content (AN) using potassium chloride solution extraction and indophenol blue colorimetry [39]. We assessed soil organic matter (organic carbon [OC]) content using the potassium dichromate volumetric method accompanied by external heating [40]. The soil microbial biomass carbon (MBC) was quantified using chloroform fumigation extraction and subsequently measured using a carbon and nitrogen analyzer [41]. pH values were determined with a pH meter.

### 2.3. DNA Extraction, Library Construction, and Metagenomic Sequencing

An E.Z.N.A.^®^ Soil DNA Kit (Omega Bio-tek, Norcross, GA, USA) was used for the extraction of genomic DNA from wheat RS samples (1 g per sample) per the protocols. To assess the concentration and purity of the extracted DNA, TBS-380 and NanoDrop2000 (Thermo Scientific Inc., Waltham, MA, USA) were utilized, respectively, on 1% agarose gel [42]. To construct a paired-end library, the DNA extract was subjected to fragmentation to an average size of nearly 400 bp through Covaris M220 (Gene Company Limited, Shanghai, China), and the library was generated with a NEXTFLEX Rapid DNA-Seq kit (Bioo Scientific in Austin, TX, USA) [43]. Adapters with complete sequencing primer hybridization sites were attached to the fragments’ blunt ends. The paired end was sequenced on an Illumina Novaseq 6000 instrument )Majorbio Bio-Pharm Technology Co., Ltd., Shanghai, China( using NovaSeq Reagent Kits and following the protocols (www.illumina.com accessed on 15 May 2024) [44]. Our sequence data were submitted to the NCBI Short Read Archive database (PRJNA1128242).

### 2.4. Sequence Quality Control and Genome Assembly

Data processing was carried out using the Majorbio Cloud Platform (www.majorbio.com accessed on 17 May 2024). Specifically, the paired-end Illumina reads were subjected to adaptor trimming and low-quality read elimination (length < 50 bp, quality value < 20, or containing N bases) via fastp [45] (https://github.com/OpenGene/fastp accessed on 19 May 2024, version 0.20.0). A collection of metagenomics data were compiled via the MEGAHIT software [46] (https://github.com/voutcn/megahit accessed on 19 May 2024, version 1.1.2), which employs succinct de Bruijn graphs. Gene prediction and annotation were performed on contigs that exceeded a length of 300 bp, chosen as the final assembly output.

### 2.5. Gene Prediction, Taxonomy, and Functional Annotation

Open reading frames (ORFs) were identified in each assembled contig using Prodigal [47] (http://compbio.ornl.gov/prodigal/ accessed on 21 May 2024) /MetaGene [48] (http://metagene.cb.k.u-tokyo.ac.jp/ accessed on 21 May 2024). According to the NCBI translation table, ORFs with a length ≥ 100 bp were selected and translated into amino acid sequences (http://www.ncbi.nlm.nih.gov/Taxonomy/taxonomyhome.html/index.cgi?chapter=tgencodes#SG1 accessed on 22 May 2024).

A non-redundant gene catalog was compiled by applying CD-HIT [49] (http://www.bioinformatics.org/cd-hit/ accessed on 23 May 2024, version 4.6.1) via a 90% sequence identity threshold and 90% coverage. This was followed by aligning high-quality reads to the non-redundant gene catalogs to determine gene abundance with a 95% identity threshold via SOAPaligner [50] (http://soap.genomics.org.cn/ accessed on 24 May 2024, version 2.21).

Using Diamond [51] (http://www.diamondsearch.org/index.php accessed on 25 May 2024, version 0.8.35) with a 1 × 10^−5^ e-value cutoff, we subjected the non-redundant gene catalog to alignment to the NR database for taxonomic annotations; an annotation of a Cluster of Orthologous Groups of proteins (COG) was carried out for the representative sequences vs. the eggNOG database; and KEGG annotation was performed through the alignment of the representative sequences to the KEGG database.

Carbohydrate-active enzyme annotation was performed using (http://hmmer.janelia.org/search/hmmscan accessed on 26 May 2024) vs. the CAZy database (http://www.cazy.org/ accessed on 26 May 2024) with a 1 × 10^−5^ e-value threshold. The antibiotic resistance annotation was performed with the Diamond vs. CARD database (https://card.mcmaster.ca/home accessed on 27 May 2024) with a 1 × 10^−5^ e-value cutoff.

### 2.6. Statistical Analysis

The Majorbio Cloud platform (https://cloud.majorbio.com accessed on 22 July 2024) was used to analyze the soil microbiota via bioinformatic analysis. α-diversity analysis was based on the reading number abundance calculation method through mothur (https://mothur.org/wiki/calculators/ accessed on 29 May 2024, version v.1.30.2). The algorithm for the corresponding index analysis was visualized using Veganv2.5-3 of R (Version 3.3.1). Using Vegan v2.5-3, a non-metric multidimensional scaling (NMDS) based on Bray–Curtis dissimilarity was used to determine the similarity between microbial communities across samples. The PERMANOVA test in the Vegan v2.5-3 package quantified the treatment’s contribution to variation.

In the results, the means are presented along with standard deviations (SDs). We calculated the significance levels for soil fertility, diversity, and richness indexes between YJ and CK groups at significance levels of *p* < 0.05 or *p* < 0.01 with a one-way ANOVA. SAS, version 9 (SAS Institute Inc., Cary, NC, USA), was used to conduct all statistical analyses.

## 3. Results

### 3.1. Soil Fertility Assessment

The soil fertility analysis showcased that the DT group displayed significantly lower total nitrogen, total phosphorus, total potassium, available phosphorus, available potassium, ammonium nitrogen, organic carbon, and microbial biomass carbon levels than the CK group. Nonetheless, nitrate nitrogen levels did not significantly differ between both groups (Table 1).

### 3.2. Sequencing Quality Assessment

After data filtering, each sample contained 92.59–124.04 million clean reads, with the average clean bases per sample exceeding 13.01 Gb. The Q20/30 values were above 97.99% and 94.28%, respectively, in both groups. The GC contents of the clean data were 63.79% and 64.31% in the DT and CK groups, respectively. The contig N50 lengths in the DT and CK groups were 522 and 559 bp, while the contig N90 lengths were 332 and 338 bp, revealing the high quality of the sequencing data (Table 2).

### 3.3. Taxonomic Levels of Microbial Community Structure

By analyzing the non-redundant gene sequences in the Nr database, 29,665 microorganism species were identified, with 26,922 species in DT soil and 27,344 in CK soil. These species were classified into 4784 genera, with 4418 genera in DT soil and 4514 in CK soil. Furthermore, the species were categorized into 1562 families (with 1434 families in DT soil and 1499 in CK soil), 815 orders (755 in DT soil and 790 in CK soil), 429 classes (411 in DT soil and 418 in CK soil), and 229 phyla (226 in DT soil and 225 in CK soil). The findings suggest that DT cropping may result in a modest reduction in microbial diversity when compared to CK cropping soil (Table 3).

### 3.4. α- and β-Diversity Assessments of Microbial Community

**Figure 1:**
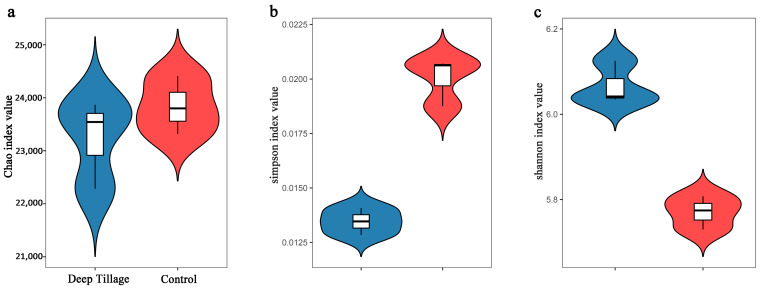
The α-diversities of deep-tillage cultivation group’s and control group’s wheat rhizosphere soil microbes. (**a**) for Chao index value, (**b**) for Simpson index value, (**c**) for shannon index value.DT and CK: Deep- and non-deep-tillage cultivated wheat rhizosphere soil groups, respectively.

The Chao index positively correlates with the number of observed species, with a higher Chao index indicating a greater number of species. The results showed no significant disparities in the Chao indexes between both groups (Figure 1a). Conversely, the Simpson index is inversely related to community diversity, with a higher Simpson index indicating lower diversity. The DT group exhibited a significantly lower Simpson index than the CK group (Figure 1b), suggesting the higher diversity of the DT group. Similarly, the Shannon index positively correlates with community diversity, with a higher Shannon index indicating greater diversity. The DT group displayed a significantly higher Shannon index than the CK group (Figure 1c), further supporting the notion of higher diversity in the DT group.

**Figure 2:**
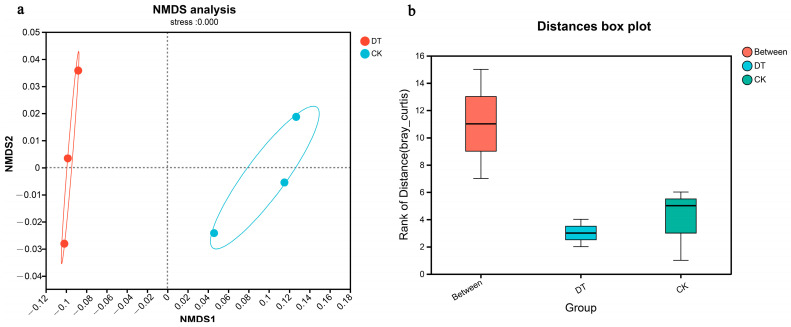
β-diversities (NMDS and ANOSIM analysis) of DT and CK cultivation wheat rhizosphere soil microbes. (**a**) for NMDS analysis, (**b**) for ANOSIM analysis. DT and CK: Deep- and non-deep-tillage cultivated wheat rhizosphere soil groups, respectively.

The NMDS analysis revealed a clearly defined and representative ordination based on the stress value. The intergroup distance exceeded that of the intragroup samples, indicating a significant degree of community aggregation and dispersion (Figure 2a). Analysis using ANOSIM further verified that the difference between both groups was more significant than within each group (Figure 2b).

### 3.5. Impacts of DT on the Rhizosphere Soil Microbial Community Structure

**Figure 3:**
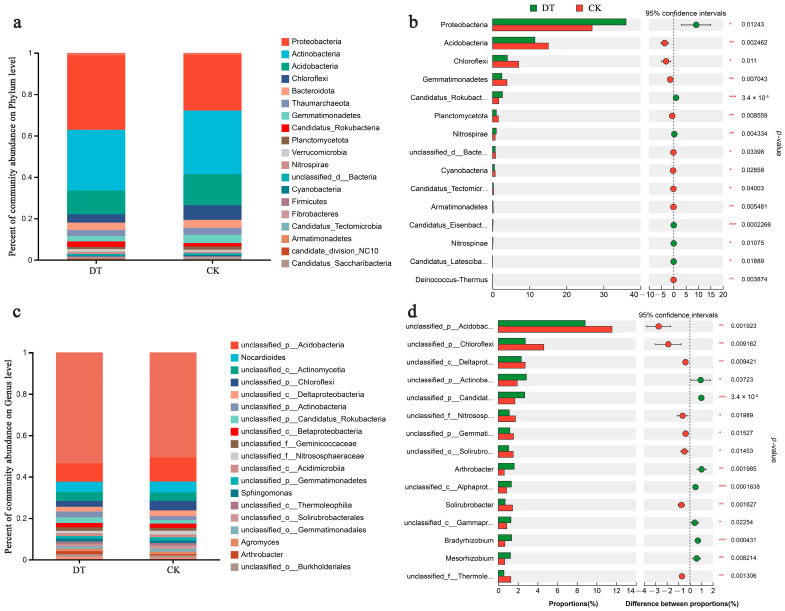
Phylum-level compositions (**a**) and differences (**b**) and genus-level compositions (**c**) and differences (**d**) in the rhizosphere soil (RS) microbiome in various cultivation modes. * represented 0.01 < *p* ≤ 0.05, ** represented 0.001 < *p* ≤ 0.01, *** represented *p* ≤ 0.001. DT and CK: Deep- and non-deep-tillage cultivated wheat RS groups, respectively.

At the phylum level, Proteobacteria, Actinobacteria, Acidobacteria, Chloroflexi, Bacteroidota, and Thaumarchaeota were the predominant RS microbes, representing more than 87% of the total abundance (Figure 3a). Specifically, the RS of DT-subjected wheat displayed significantly higher Proteobacteria abundance but significantly lower Acidobacteria and Chloroflexi abundances than the CK group (both *p* < 0.05, Figure 3b).

At the class level, Actinomycetia, Alphaproteobacteria, Gammaproteobacteria, Betaproteobacteria, Thermoleophilia, and Deltaproteobacteria were the predominant microbes, representing over 60% of the total abundance of classes (Figure S1a). Specifically, Alphaproteobacteria, Gammaproteobacteria, and Betaproteobacteria exhibited significant abundances in the RS of DT cultivation wheat, unlike the CK group (*p* < 0.05). In addition, the RS of DT-cultivated wheat exhibited significantly higher Thermoleophilia abundance than the CK group (*p* < 0.05, Figure S1a).

At the taxonomic order level, Hyphomicrobiales, Propionibacteriales, Micrococcales, Rhodospirillales, and Burkholderiales were identified as the dominant microbes (Figure S1c). Specifically, the RS of wheat under DT cultivation had significantly higher Hyphomicrobiales, Micrococcales, and Burkholderiales abundances (*p* < 0.05) and significantly lower Solirubrobacterales abundance than the CK group (*p* < 0.01, Figure S1d).

At the family level, the prominent microbial taxa observed included Nocardioidaceae, Xanthomonadaceae, Nitrososphaeraceae, and Microbacteriaceae (Figure S1e). Specifically, the DT group displayed significantly greater relative Xanthomonadaceae and Microbacteriaceae abundances (*p* < 0.05) and a significantly lower Solirubrobacteraceae proportion than the CK group *(p* < 0.01, Figure S1f).

Figure 3c depicts the genus-level RS microbiome composition in both groups. Dominant microbes at the genus level included *Nocardioides*, *Sphingomonas*, *Agromyces*, and *Arthrobacter*. The DT group displayed significantly lower unclassified_p__Acidobacteria, unclassified_p__Chloroflexi, and unclassified_c__Deltaproteobacteria abundances than the CK group (*p* < 0.01). Conversely, the DT group had significantly higher unclassified_p__Actinobacteria and unclassified_p__Candidatus_Rokubacteria abundances than the CK group (*p* < 0.05, Figure 3d).

At the taxonomic species level, the dominant microbes identified in this study included *Acidobacteria_bacterium*, *Actinomycetia_bacterium*, *Chloroflexi_bacterium*, *Deltaproteobacteria_bacterium*, *Actinobacteria_bacterium*, *Candidatus_Rokubacteria_bacterium*, and *Geminicoccaceae_bacterium* (Figure S1g). Specifically, the DT group had significantly lower *Acidobacteria_bacterium*, *Chloroflexi_bacterium*, and *Deltaproteobacteria_bacterium* abundances (*p* < 0.01) and significantly higher *Actinobacteria_bacterium* and *Candidatus_Rokubacteria_bacterium* abundances than the CK group (*p* < 0.05, Figure S1h).

### 3.6. Impacts of DT on RS Microbial Function Differences

**Figure 4:**
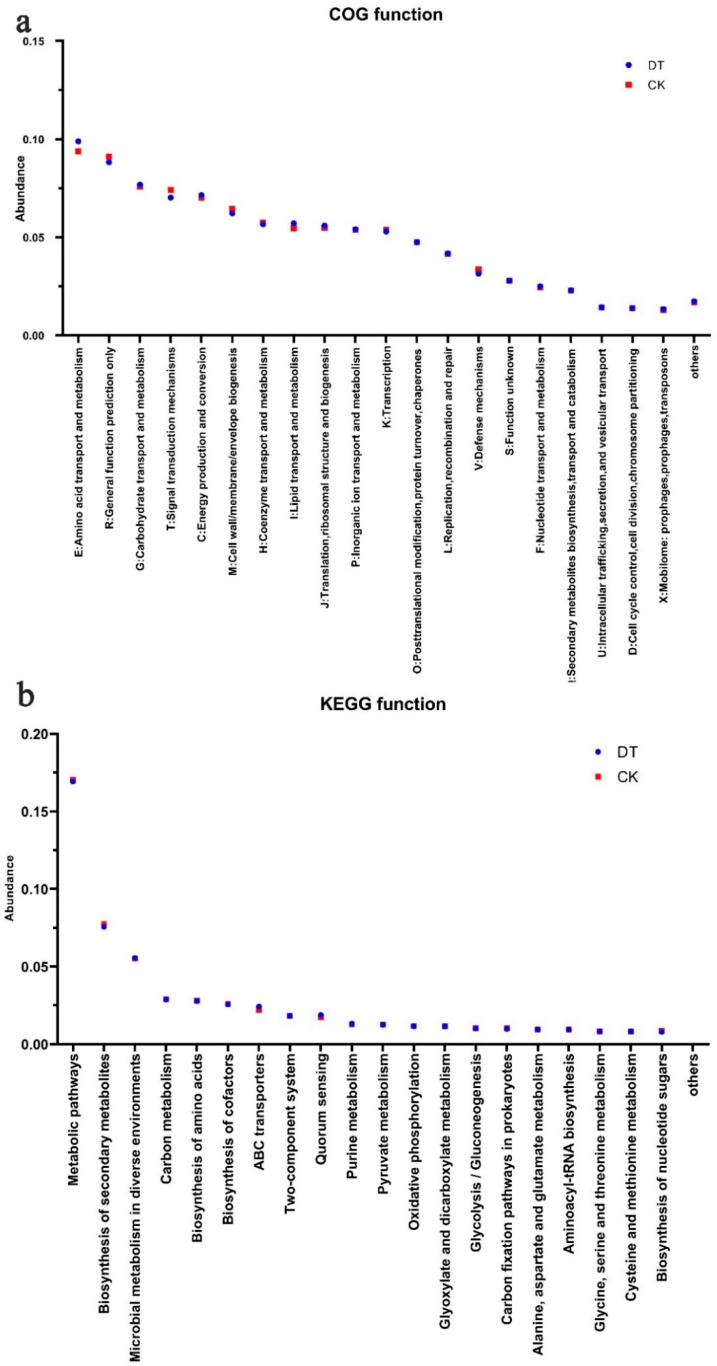
Gene abundance in microbes of deep- and non-deep-tillage rhizosphere soil. (**a**) Relative abundance changes in COG genes; (**b**) KEGG metabolic pathway-related functional genes.

According to the functional analysis of the COG, genes correlated with carbohydrate, amino acid, and lipid transport and metabolism were significantly elevated in the RS of DT-subjected wheat. Conversely, genes related to signal transduction mechanisms and defense mechanisms experienced a significant decrease in the DT group (Figure 4a). Meanwhile, the KEGG functional annotation analysis revealed that the DNA sequences enriched in metabolic pathways and the biosynthesis of secondary metabolites and nucleotide sugars were significantly lower in the DT group than in the CK group. Conversely, DNA sequences enriched in ABC transporters, quorum sensing, and purine metabolism displayed a significant elevation in the DT group in comparison to the CK group (Figure 4b). These findings align with the COG functional analysis results.

### 3.7. Effects of DT on RS Microbial Carbohydrate Enzyme Enrichment in Wheat Cultivation

**Figure 5:**
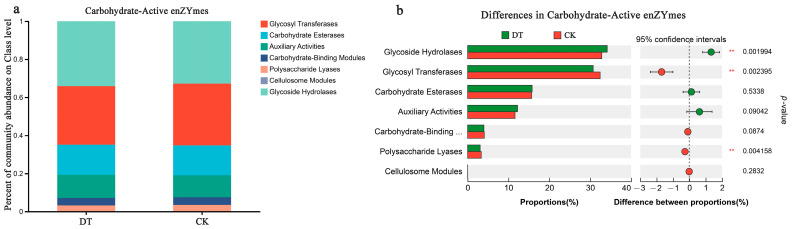
Carbohydrate enzyme-related genes in DT and CK rhizosphere soil (RS) microbes. (**a**) Proportion of the carbohydrate enzyme-correlated genes in the RS of both groups; (**b**) comparison of the difference in carbohydrate enzyme-correlated genes in the RS microbes of both groups. ** represented 0.001 < *p* ≤ 0.01. DT and CK: Deep- and non-deep-tillage cultivated wheat RS groups, respectively.

The functional annotation information for carbohydrate-active enzyme genes was obtained from the CAZy database. Glycoside hydrolases (GHs) were the most abundant in the DT and CK groups, representing 34.17% and 32.84%, respectively (Figure 5a). There was a statistically significant difference (*p* < 0.01) between both groups, with the DT group displaying a higher abundance than the CK group (Figure 5b). The relative Glycosyl Transferase (GT) abundance was 30.73% and 32.43% in the DT and CK groups, respectively, following the GHs. Conversely, the DT group showed significantly lower GT content (*p* < 0.01) than the CK group. Similarly, the DT group showed a significantly reduced relative abundance of Polysaccharide Lyases, 3.07% (*p* < 0.01), compared with the CK group. Carbohydrate esterase prevalence in the DT group was 15.79%, followed by 12.22% Auxiliary Activities, 3.99% Carbohydrate-Binding Modules, and 0.01% Cellulosome Modules, showing no statistically significant variance in comparison to the CK group.

### 3.8. Impacts of DT on RS Microbial Antibiotic Resistance Ontology (ARO) Enrichment in Wheat Cultivation

**Figure 6:**
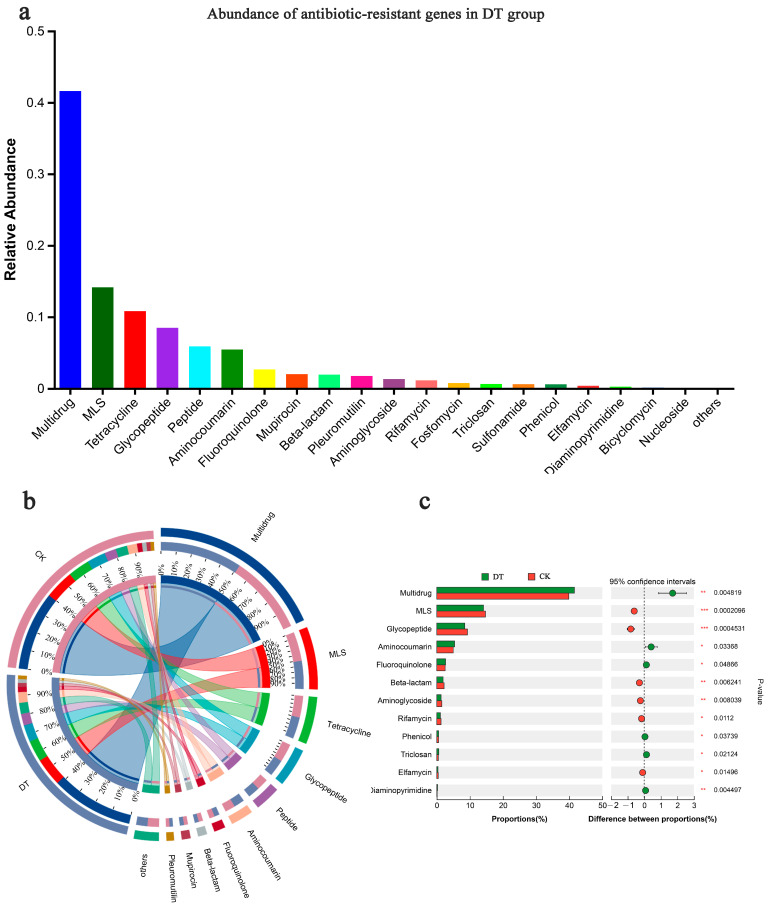
Antibiotic resistance ontology (ARO) composition and abundance. (**a**) ARO abundance in the rhizosphere soil of deep-tillage cultivated wheat; (**b**) ARO composition and (**c**) difference in both groups. * represented 0.01 < *p* ≤ 0.05, ** represented 0.001 < *p* ≤ 0.01, *** represented *p* ≤ 0.001. DT and CK: Deep- and non-deep-tillage cultivated wheat RS groups, respectively.

The ARO was categorized into various CARD antibiotic classes, with significant enrichments shown in the multidrug, MLS, tetracycline, and glycopeptide classes (Figure 6a). Among these, multidrug antibiotics exhibited the highest abundance in both groups (Figure 6b) and a significant disparity (*p* < 0.01) between both groups, with the DT group showing a larger abundance. Following multidrug antibiotics, the DT group showcased significantly lower macrolide antibiotic (MLS) abundance than the CK group (*p* < 0.01), with a similar trend observed for glycopeptide antibiotics (Figure 6c). A comprehensive examination showed that the relative abundances of the multidrug and aminocoumarin classes were significantly increased in the RS of the DT group. Conversely, the relative abundances of the glycopeptide, beta-lactam, and aminoglycoside classes were significantly decreased in the DT group.

## 4. Discussion

The widely used agricultural method of reintroducing straw into the field, combined with DT, has been shown to successfully reduce soil compaction and increase the depth of the cultivation layer [52]. Moreover, DT has been shown to improve agricultural productivity by moving straw and fertilizer from the uppermost layer into the lowermost soil layers, therefore acting as a nutrient supply for the deep soil layer and crops [53]. An analysis of the soil fertility indicates that the DT cultivation of wheat results in lower fertility levels in the 0–20 cm soil layer than rotary tillage cultivation (CK). This aligns with previous research indicating that utilizing CK throughout the wheat season leads to increased soil nutrients within the 0–20 cm soil layer, whereas utilizing DT within the 20–40 cm soil layer results in higher soil nutrient levels [54]. CK culture boosts the dense accumulation of plant wastes, fertilizers, straw, and other nutrients on the soil surface, therefore improving the accessibility of nutrients in the upper layers of the soil [55].

The α-diversity Simpson and Shannon indexes indicated that the microbial community in the RS of DT-subjected wheat exhibited higher diversity compared to wheat subjected to CK cultivation. Furthermore, an analysis of β-diversity using NMDS and ANOSIM revealed a significant difference between the two cultivation methods, surpassing the differences observed within each group. These findings align with previous research on DT cultivation [6,18], suggesting the positive impact of DT cultivation on microbial diversity in wheat RS.

Within the realm of functional annotation classification, the majority of sequences were enriched in categories related to metabolism function, particularly amino acid/carbohydrate/vitamin/cofactor/energy/nucleotide metabolism. Amino acid, carbohydrate, and energy metabolism are vital for wheat growth, development, and quality. This study showed that there was a higher abundance of genes linked with carbohydrate/amino acid/energy metabolism in the RS microorganisms of DT wheat. Our COG function analysis indicates that amino acid and carbohydrate transport and metabolism, in addition to energy production and conversion functions, are more pronounced in the RS of wheat cultivated using DT methods compared to CK methods. Similarly, the KEGG function analysis reveals that microbial metabolism in diverse environments is more active when DT is used to cultivate wheat RS than when CK is used. Specifically, the carbohydrate metabolism function, which includes the synthesis of starch and sucrose, plays a crucial role in influencing wheat yield [56]. The metabolism of amino acids is crucial for facilitating plant signaling and metabolic adjustments in response to energy deficiency induced by stress [57].

Proteobacteria, Actinobacteria, and Acidobacteria were identified as the predominant bacterial taxa in the RS of wheat, aligning with prior research findings [58,59]. A notable association was observed between Proteobacteria and various nitrogen cycle-related metabolisms, indicating their involvement in soil nitrogen transformation [60]. Furthermore, a higher abundance of Proteobacteria was detected in carbon-rich soils through a comprehensive analysis of the soil microbial community [61], underscoring their direct participation in the soil carbon cycle [62]. Organisms belonging to the Acidobacteria phylum exhibit a wide distribution across various ecosystems, with a significant abundance of genes implicated in nitrogen, carbon, and sulfur metabolic pathways [63].

At the family level, the DT group showed a significantly greater relative abundance of Microbacteriaceae compared to the CK group. The presence of Microbacteriaceae in the rhizosphere bacterial network map suggests a notable and positive relationship with the soil-soluble organic nitrogen conversion rate [64]. At the genus level, the DT group displayed a significantly higher relative abundance of Arthrobacter compared to the CK group. Certain members of the Arthrobacter genus in soil have been associated with the degradation of phenolic acids and heavy metals [65,66]. Furthermore, DT cultivation significantly increased the relative abundances of *Bradyrhizobiaceae* and *Mesorhizobium*. According to Jiang [67], *Bradyrhizobiaceae* has been shown to improve soybean tolerance to atrazine through the modulation of the RS microbial community and gene expression associated with amino acid and carbohydrate metabolism. This suggests that DT of wheat modifies the RS microbial community composition and activity, leading to enhanced nutrient availability for wheat, increased nitrogen uptake by wheat roots, and subsequent conversion into amino acids.

Carbon serves as the primary substrate for amino acid biosynthesis, with certain genes that contribute to carbohydrate transport and metabolism implicated in this process [68]. CAZyme coding genes change as a result of soil properties exerting selective pressure on soil microorganisms [69]. Our investigation revealed a notable increase in the abundance of GHs following DT cultivation. These enzymes, commonly referred to as glycosidases, are essential catalysts present in various organisms, crucial for carbohydrate metabolism through the cleavage of glycosidic bonds in complex sugars [70,71]. Our COG functional analysis of the soil microbiome in response to DT cultivation revealed an increase in the relative abundance of genes correlated with carbohydrate and energy metabolism. This shift in microbial community structure influences carbohydrate and amino acid metabolism, ultimately enhancing the wheat crop’s quality.

Antibiotic resistance genes could be detected in various environments such as animal feces and soil [72]. The emergence of antibiotic resistance genes (ARGs) in microorganisms predates the human use of antibiotics [73]. Pervasive antibiotic use is the most important factor for the global dispersal of ARGs; soil ARGs can accumulate with repeated applications of animal manures with antibiotics [74]. Studies have shown that soil is a major repository of antibiotic resistance genes; the soil bacterial community is a rich source of mobile antibiotic resistance genes [75,76]. The prevalence of antibiotic resistance genes in the RS of wheat cultivated using DT was significantly greater than that cultivated with CK. A high abundance of multidrug efflux pumps has been reported in soil bacteria, significantly contributing to bacterial adaptation and survival in soil environments [77]. These efflux pumps are not only involved in expelling antibiotic compounds but also in the removal of endogenous metabolites, heavy metals, and organic pollutants [78]. Studies revealed that soil microbial community was significantly correlated with ARG hosts, suggesting that fertilizers affect soil ARGs abundance mainly by altering soil microbial composition [79]. The abundance and diversity of ARGs in the soil decreased incrementally with the reduction in the application ratio of organic fertilizers [80,81]. In our study, the deep tillage cultivation mode significantly changed soil fertility, which in turn affected microbial community structure and significantly altered the abundance of antibiotic resistance genes.

Metagenomics was deployed to investigate the DT impact on the microbial composition within the RS of wheat. Our findings indicated that DT influenced the microbial community structure, notably enhancing populations of nitrogen-fixing bacteria, particularly Acidobacteria. Furthermore, there was an observed increase in the abundance of genes related to carbohydrate biosynthesis and amino acid metabolism within the soil microbial community of DT wheat, both of which are crucial pathways for wheat productivity. The current study offers indispensable insights for enhancing the sustainability and productivity of wheat ecosystems.

## Figures and Tables

**Table 1:** Soil fertility attributes of deep tillage and control groups.

	Deep Tillage Group	Control Group
Total Nitrogen g/kg	0.73 ± 0.08 ^b^	1.42 ± 0.069 ^a^
Total Phosphorus g/kg	0.73 ± 0.07 ^b^	1.21 ± 0.13 ^b^
Total Potassium g/kg	19.60 ± 1.10 ^b^	21.63 ± 0.23 ^a^
Available Phosphorus mg/kg	11.47 ± 1.17 ^b^	20.12 ± 2.31 ^a^
Available Potassium mg/kg	91.48 ± 12.65 ^b^	200.13 ± 37.65 ^a^
Nitrate Nitrogen mg/kg	29.17 ± 11.70 ^a^	30.44 ± 3.60 ^a^
Ammonium Nitrogen mg/kg	9.94 ± 0.85 ^b^	16.00 ± 3.37 ^a^
Organic Carbon g/kg	10.78 ± 1.58 ^b^	25.47 ± 0.74 ^a^
Microbial Biomass Carbon mg/kg	213.28 ± 105.74 ^b^	580.72 ± 128.18 ^a^
pH	5.7 ± 0.20 ^a^	6.1 ± 0.12 ^a^

Data represent mean ± standard error (SE), with lowercase superscript letters within the same row indicating statistical significance at *p* < 0.05.

**Table 2:** Sequencing data evaluation of deep- and non-deep-tillage rhizosphere soil microbes.

Sample	Deep Tillage	Control
Raw reads	111,538,338 ± 19,857,435	114,697,586 ± 12,431,916
Raw base (bp)	16,842,289,038 ± 1,877,219,439	17,319,335,587 ± 2,998,472,743
Clean reads	110,581,845 ± 12,301,636	115,381,894 ± 19,929,484
Clean base (bp)	16,684,253,472 ± 1,856,544,034	17,409,238,514 ± 3,007,507,178
Percent in raw reads	99.14 ± 0.04	99.04 ± 0.25
Percent in raw bases	99.06 ± 0.04	98.96 ± 0.25
Clean Q20 (%)	98.12 ± 0.05	97.99 ± 0.32
Clean Q30 (%)	94.63 ± 0.10	94.28 ± 0.78
Clean GC content (%)	63.79 ± 0.14	64.31 ± 0.10
N50 (bp)	522 ± 11.02	559 ± 17.24
N90(bp)	332 ± 1.15	338 ± 2.31

**Table 3:** Taxonomic levels of recognized microorganisms in deep- and non-deep-tillage rhizosphere soil.

	Phylum	Class	Order	Family	Genus	Species
Deep Tillage	226	411	755	1434	4418	26,922
Control	225	418	790	1499	4514	27,344
Common	222	400	730	1371	4148	24,601
Total	229	429	815	1562	4784	29,665

## Data Availability

We submitted the raw data to the NCBI database BioProject (PRJNA1128242).

## Supplementary Materials

The following supporting information can be downloaded at: https://www.mdpi.com/article/10.3390/microorganisms12112339/s1, Figure S1: Class-level compositions (a) and differences (b), order-level compositions (c) and differences (d), family-level compositions (e) and differences (f), and species-level compositions (g) and differences (h) in the rhizosphere soil (RS) microbiome in various cultivation modes. * represented 0.01 < *p* ≤ 0.05, ** represented 0.001 < *p* ≤ 0.01, *** represented *p* ≤ 0.001. DT and CK: Deep- and non-deep-tillage cultivated wheat RS groups, respectively.
